# Supplementary material for: A novel mouse model for studying complications related to type 2 diabetes using a medium-fat diet, fructose, and streptozotocin
Source: Sci Rep. 2025 Jul 1;15:20861. doi: 10.1038/s41598-025-04335-3 (PMC12217141; doi:10.1038/s41598-025-04335-3)
Supplement: Supplementary file 2 — Supplementary Material 2 [file 41598_2025_4335_MOESM2_ESM.docx]

| ***Table 1 Supplementary Material*** | | |
| --- | --- | --- |
| ***Composition of the Solid Diets Used (calculated in 100g)*** | | |
|  | ***Dieta Control*** | ***MFD*** |
| **Moisture (max)** | 12,0% | 10,0% |
| **Proteins (min)** | 23,0% | 28,0% |
| **Carbohydrates*** | 50,0% | 39,5% |
| *Crude Fiber (max)* | 6,0% | 3,5% |
| **Fat (min)** | 5,0% | 15,0% |
| **Total minerals** | 10,0% | 7,5% |
| *Calcium (min. - max.)* | 1% - 1,4% | 1,5% - 1,7% |
| *Phosphorus (min. - max.)* | 0,5% - 0,8% | 1,1% - 1,4% |

**Online Supplemental Materials**

***Supplementary Figure 1. Analysis of urinary parameters during an 18-hour time window utilizing a metabolic cage. (A) urine output volume, (B) glomerular filtration rate (GFR), (C) urinary albumin, (D) urinary creatinine, (E) total urinary protein, (F) urinary urea, (G) UACR was calculated as the ratio between urinary albumin concentration (mg) and urinary creatinine concentration (g), (H) plasma urea, (I) plasma creatinine. N (n= 5), D (n= 5), D+T (n=5). (*) p ≤ 0.05; (**) p< 0.01; ns: non-significant.***

**A**

**B**

**C**

**D**

**E**

**F**

**G**

**H**

**I**
